# MiR-486 Regulates Lactation and Targets the PTEN Gene in Cow Mammary Glands

**DOI:** 10.1371/journal.pone.0118284

**Published:** 2015-03-04

**Authors:** Dan Li, Xuejiao Xie, Jie Wang, Yanjie Bian, Qingzhang Li, Xuejun Gao, Chunmei Wang

**Affiliations:** Key Laboratory of Dairy Science of Education Ministry, Northeast Agricultural University, Harbin 150030, Heilongjiang, China; Northwestern University, UNITED STATES

## Abstract

Mammary gland development is controlled by several genes. Although miRNAs have been implicated in mammary gland function, the mechanism by which miR-486 regulates mammary gland development and lactation remains unclear. We investigated miR-486 expression in cow mammary gland using qRT-PCR and ISH and show that miR-486 expression was higher during the high-quality lactation period. We found that miR-486 targets phosphoinositide signaling in the cow mammary gland by directly downregulating PTEN gene expression and by altering the expression of downstream genes that are important for the function of the mammary gland, such as AKT, mTOR. We analyzed the effect of β-casein, lactose and triglyceride secretion in bovine mammary gland epithelial cells (BMECs) transfected by an inhibitor and by mimics of miR-486. Our results identify miR-486 as a downstream regulator of PTEN that is required for the development of the cow mammary gland.

## Introduction

Bovine mammary glands arise from the ectoderm during embryonic development and continue to develop postnatally through puberty, pregnancy, lactation, and subsequent involution. Most developmental and functional differentiation in the mammary gland occurs after the birth of offspring [1]. During lactation, the mammary gland secretes milk, which provides nearly all the nutrient requirements of the newborn offspring during the transition from pregnancy to lactation [2]. The gland develops primarily postnatally, and its development is mainly controlled by steroids, peptide hormones, and cell matrix interactions during different stages. Several pathways have been shown to modulate the progression of mammary gland development. Additionally, more than 100 genes have been shown to modulate various aspects of mammary physiology, from the formation of the fetus to remodeling of the gland during involution [2–3]. MicroRNAs (miRNAs) have also been shown to regulate cell processes, and many miRNAs are involved in mammary gland development and tumorigenesis [4].

Due to the unique developmental features found during distinct stages of lactation, the mammary gland represents an important model for use in studies to elucidate signaling related to cell cycle progression, survival, proliferation, differentiation, and cell death. Despite the relatively recent recognition of miRNAs as key regulators of cellular function, little research has focused of the function of miRNAs during normal mammary development, and even less research has focused the role of these compounds during bovine mammary gland development. The biological roles remain unclear between miRNAs and genes that associated with the transcriptional modulation in the dairy cow mammary gland during lactation [5].

MiRNAs play a key role in regulating a variety of cellular processes by repressing messenger RNA (mRNA) targets, and many studies have shown that miRNAs modulate intracellular signaling pathways that are involved in apoptosis, metabolism, cell proliferation, and cell growth. Some studies have shown that miRNAs are associated with the modulation of important physiological processes, such as cellular proliferation, lipid metabolism, and innate immunity in dairy cow mammary gland tissues during puberty, pregnancy, lactation, and post-lactation. Ahmet [6] shows that the miR-212/132 family of miRNAs is essential to the epithelial-stromal interaction during mouse mammary gland development, and this family specifically modulates the stroma rather than the epithelial tissue. Overexpression of certain miRNAs, such as miR-101a, miR-126–3p, and miR-15a, suppresses mammary gland epithelial cell differentiation in mice, thus inhibiting mammary gland differentiation [7–9]. Although some researchers have studied normal gland biology, almost all studies of miRNAs expression during the various phases of lactation have been conducted only in mice [10]. However, direct evidence of the suppression of mammary gland epithelial cell differentiation by specific miRNAs is still lacking.

The expression of miR-486 has been shown to affect various processes. For example, miR-486 represses the development of pancreatic ductal adenocarcinomas by inhibiting the expression the gene CD40 [11], inhibits SIRT1 deacetylase activity in human adipose tissue-derived mesenchymal stem cells [12], stimulates muscle myoblast differentiation by downregulating Pax7 [13], and downregulating PTEN (phosphatase and tensin homolog) and Foxo1a in muscle cells [14]. PTEN is a protein and lipid phosphatase. The mutation of PTEN is a key step in the development of a variety of human tumors, including breast, brain, prostate, and endometrium tumors [15]. Additionally, PTEN has been found to modulate various normal cellular processes, such as proliferation, cell adhesion, migration, and apoptosis [16]. In addition, PTEN overexpression in mice decreases the proliferation of mammary epithelium, increases cell apoptosis, and reduces the differentiation of mammary epithelial cells, resulting in the death or growth postponement of newborn offspring [17]. PTEN, is a downregulator of the PI3K/AKT signaling pathway as it dephosphorylates phosphatidylinositol 3, 4, 5-phosphate, the product of PI3K activity.

We were interested in miRNAs that target regulators with potential effects on mammary development, particularly those that might regulate milk secretion, such as PTEN. In this report, we show that the function of miR-486 is indispensable for regulating PTEN in bovine mammary epithelium. We confirmed that miR-486 is expressed in both bovine mammary gland tissues and in mammary epithelial cells *in vitro*.

## Materials and Methods

### Ethics statement

All animals received humane care as outlined in the Guide for the Care and Use of Experimental Animals of the National Institutes of Health. All experimental procedures with animals used in the present study had been given prior approval by the Northeast Agricultural University Provincial Experimental Animal Manage Committee. All surgery was performed to minimize suffering.

### Animals, tissue sampling, cell culture, and transfection

Nine healthy multiparous Holstein cows were obtained from the Holstein Cattle Association of Australia, and mammary glands were collected from these animals. The cows weighed an average (mean s.e.) of 609±9.08 kg with a parity of 3.1±0.19. The animals were separated into 3 groups by developmental stage: the lactation with high quality milk stage (milk yield 30.6±0.78 kg/d, milk protein >3.0%, and milk fat >3.5%; n = 3), the lactation with poor quality milk stage (milk yield 30.6±0.78 kg/d, milk protein <3.0%, and milk fat <3.5%; n = 3), and the pregnancy stage (n = 3). All of the cows were fed with standard foodstuffs consisting of 30% roughage and 70% concentrate. The cows were slaughtered by exsanguinations, and their mammary tissue was aseptically excised 5 cm from the base of the healthy breast and 3 cm from the midline that divides the core of the secretory gland tissue. After removing the connective tissue, the remaining tissue was cut into small blocks with a thickness of 1 cm. The mammary tissue samples were immediately frozen in liquid nitrogen and stored at-80°C for later analysis. All animal experiments were approved by the Institutional Animal Care and Use Ethics Committee of Northeast Agricultural University and conducted in accordance with the Guidelines for Experimental Animals from the Ministry of Science and Technology (Beijing, China).

Bovine mammary gland epithelial cells (BMECs) were cultured in DF-12 growth medium (a 1:1 mixture of Ham’s F-12 and Dulbecco’s modified eagle medium) (Gibco, Grand Island, NY, USA) supplemented with 10% fetal bovine serum (FBS) (Gibco, Grand Island, NY, USA) and incubated at 37°C in a humidified atmosphere of 5% CO_2_.

The indicated cells were transfected with 50 nmol miR-486 mimics, inhibitors, or the corresponding negative control constructs (miR-NC, Anti-NC) (GenePharma, Shanghai, China) using Lipofectamine 2000 (Invitrogen Life Technologies, Carlsbad, CA, USA) according to the manufacturer’s instructions, respectively. Twenty-four hours after transfection, the cells were treated with cell differentiation medium.

### Small RNA sequencing

We extracted total RNA from mammary gland tissues at 3 different developmental stages using Trizol reagent (Invitrogen, Carlsbad, CA, USA) according to the manufacturer’s instructions. The RNA samples were sent to the Beijing Genomics Institute (Shenzhen, Guangdong, China), where a small RNA library was constructed and sequenced using a Genome Analyzer (Illumina, San Diego, USA). All of the data were stored in the Short Archive section of the NCBI GEO under accession number GSE57991. The sequencing data were first filtered into mRNA using the Rfam 10.1 and Genebank databases and then mapped to miRBase 18. The mapped data were used to identify significant differences in miRNA expression [18].

### Plasmid Construction

There are two predicted binding miR-486 regions of the 3′UTR of PTEN (Cow PTEN NM_000314). Therefore, we decided to synthesize the following sequences. Both wild-type (wt) and mutant (mu) PTEN 3′UTRs were constructed by Biomics Biotechnologies. The wild-type 3′UTR sequence of PTEN was 5′-CTAGA GAAGTGAATCTGTATTGGG**GTACAGGA**ATGAAAAAATATGATGT**GTACAGGA**TAATT-3′, and mutant 3′UTR sequence of PTEN is 5′-CTAGA GAAGTGAATCTGTATTGGG**GAGGCATA**ATGAAAAAATATGATGT**ATGAGCAG**TAATT-3′. The wild-type and mutant PTEN 3′UTRs were cloned into the Xbal site of the pGL3-Control vector (Promega Madison, WI, USA), and the resulting vectors were named pGL3/PTEN(pGL3-wt) and pGL3/PTEN/mut(pGL3-mu), respectively.

### Luciferase activity assay

HEK-293 cells were cultured in 24-well plates, and each plate was transfected with 80 ng of pGL3/PTEN vector or pGL3/PTEN/mut vector containing firefly luciferase and 4 ng of the pRL-TK vector (Promega Madison, WI, USA) containing 4 pmol miR-486 mimics or negative control. The cells were transfected using Lipofectamine 2000 (Invitrogen). After 48 h of transfection, relative luciferase activity was calculated by normalizing the firefly luminescence to the renilla luminescence using the Dual-Luciferase Reporter Assay (Promega Madison, WI, USA) according to the manufacturer’s instructions. The experiments were performed in triplicate.

### Quantitative polymerase chain reaction (qPCR) assays

After the transfection of the cells with the miR-486 mimics/inhibitors, total RNA was purified using Trizol reagent (Invitrogen) following the manufacturer’s instructions. cDNA (complementary DNA) was synthesized using the PrimerScript RT-PCR kit (TaKaRa) according to the manufacturer’s instructions.

The expression of miR-486 and the 5S internal control gene were quantified using real-time PCR quantification and the Hairpin-it miRNAs qPCR Quantitation Kit (GenePharma, Shanghai, China) according to the manufacturer’s protocol. Specific primers for miR-486 and 5S were designed by GenePharma. The expression of these genes was analyzed using a 7300 Fast Real-Time PCR System (Applied Biosystems, Foster City, CA, USA).

The expression of PTEN, AKT, mTOR, and β-actin was quantified using Real-Time-PCR quantification and SYBR Premix Ex Taq II (TaKaRa) according to the manufacturer’s instructions. β-actin was used as a loading control. The primers that were used for qRT-PCR are listed in Table 1. All qPCR reactions were performed in triplicate.

**Table 1:** Primers of genes for fluorescence-based quantitative real-time PCR.

Gene	Primer Sequence (5′–3′)	Amplicon size
PTEN	F:CACCTATTCCTCAGCCCTTAT	273bp
	R:AACCCTCATTCAGACCTTCAC	
mTOR	F:ATGCTGTCCCTGGTCCTTATG	178bp
	R:GGGTCAGAGAGTGGCCTTCAA	
AKT	F:TAAAGAAGGAGGTCATCGTGG	181bp
	R:CGGGACAGGTGGAAGAAAA	
β-actin	F:AAGGACCTCTACGCCAACACG	249bp
	R:TTTGCGGTGGACGATGGAG	

### *In Situ* hybridization

*In situ* hybridization (ISH) experiments with 5-μm paraffin sections of bovine mammary gland tissues were performed using a DIG-labeled locked nucleic acid probe against miR-486 (5-CTCGGGGCAGCTCAGTACAGGA-3), used scramble (Exiqon, USA) as the negative control probe. Tissues were dewaxed, postfixed in 4% paraformaldehyde (PFA) for 1 h, and digested in proteinase K solution (5 μg/ml) at 37°C for 5 min. The slides were pre-hybridized at 37°C for 1 h in 50% formamide. The LNA probe was diluted with ISH buffer (1.5 pM), and the probe was denatured at 95°C for 8 min. Hybridization was performed overnight at 55°C, followed by post-hybridization washes in 5×SSC and 0.2×SSC at 60°C. After exposure for 10 min to 3% H_2_O_2_, the slides were incubated with a mouse monoclonal antibody against digoxin (Abcam Technology, Cambridge, MA, USA) at 37°C for 30 min and then incubated with an HRP-conjugated goat-anti-murine secondary antibody (Abcam). After antibody treatment, the sections were incubated with 0.04% DAB + 0.03% H_2_O_2_ to develop the color for 30 min, after which the sections were counterstained slightly with Mayer’s hematoxylin. An Olympus CX31 microscope was used.

### Western blot analysis

After the overexpression and inhibition of miR-486, the cell lysates were separated on a 10% SDS-PAGE gel (30 μg protein per sample), and the proteins were then transferred onto nitrocellulose membranes (Bio-Rad, Shanghai, China). Nonspecific binding sites were blocked using a 5% skim milk solution for 1 h at 37°C, and the membranes were then incubated with a rabbit polyclonal antibody against PTEN (Santa Cruz Biotechnology Inc., Dallas, TX, USA), AKT, phospho-AKT (Ser473), MTOR (Cell Signaling Technology, Beverly, MA, USA), or phospho-MTOR (S2448) or a murine polyclonal antibody against β-actin (Santa Cruz Biotechnology Inc.) overnight at 4°C; this was followed by incubation with goat-anti-rabbit or goat-anti-murine secondary antibodies conjugated to HRP (Zhongshan-Bio, Beijing, China), and the proteins were visualized using Super ECL Plus (ApplyGEN, Beijing, China). β-actin was used as a loading control.

### Immunofluorescence

BMECs transfected with mimics, NC (negative control), and inhibitors were cultured on glass-bottomed cell culture dishes (NEST, 801002); then, the liquid was removed gently, and the cells were rinsed twice with TBSTx (TBS supplemented with 0.1% TritonX-100). The cells were fixed in ice-cold 4% paraformaldehyde for 10 min and then washed with TBSTx 3×5 min. The cells were incubated with TBSTx containing 5% BSA (bovine serum albumin) (TBSTx-5% BSA) and then incubated with the PTEN antibody (Santa Cruz Biotechnology) diluted 1:50 with TBSTx-5% BSA, and finally incubated with 1:200 FITC-conjugated goat anti-rabbit IgG secondary antibodies (ZSGB-BIO, Beijing, China); all antibodies were incubated with the cells for 1 h at 37°C, and all wash steps involved TBSTx 3×5 min. The plates were stained with propidium iodide (PI) (Roche, Florence, SC, USA). Images were captured using a confocal laser scanning microscope (Leica TCS SP2 AOBS, Germany).

### Cell cycle analysis

Cell cycle analysis was performed using flow cytometry (FCM) as follows: harvested cells were washed with cold PBS and fixed with 70% ethanol overnight at 4°C, and washed with cold PBS. The cells were then incubated with PI (50 μg/ml) and mixed with 2 μl/ml TritonX-100 for 20 min at room temperature in the dark. The cells were then suspended in 500 μl PBS. Samples were analyzed using flow cytometry on a Cytomics TMFC500 flow cytometer, and the data were analyzed using Mod Fit LT 3.2 software (Verity Software House, USA). The FCM analysis was carried out in triplicate.

### Cell Proliferation

Cell proliferation was determined using the colorimetric water-soluble tetrazolium salt (WST-8) assay using a Cell Counting Kit-8 (Dojindo Molecular Technologies, Inc., Japan) according to the manufacturer’s instructions. After 24 h of transfection with miR-486 mimics/inhibitors, BMECs were seeded onto a 96-well plate (4×10^4^ cells per 100 μl per well), and 10 μl of CCK-8 solution was added to each well. Cells were incubated for 2 h, and cell proliferation was documented. Absorbance was measured at 450 nm using a microplate reader with a reference wavelength of 650 nm. Averages of 6 replicates were analyzed, and statistical analysis was performed using the t-test.

### Cell proliferation assay

BMECs were seeded in glass-bottomed cell culture dishes. After 48 h of transfection, cell proliferation was quantified based on the incorporation of 5-ethynyl-2-deoxyuridine (EdU) into DNA using a Cell-Light EdU Apollo 488 *In Vitro* Imaging kit (Ribobio, Guangzhou, China) according to the manufacturer’s instructions. Images were taken and analyzed using a confocal laser scanning microscope. The cell proliferation rate was calculated as the percentage of EdU-positive nuclei compared to Hoechst-stained nuclei, and more than 5 fields were captured per well. Image-Pro Plus (IPP) 6.0 software (Media Cybernetics Inc., Bethesda, MD, USA) was used to detect the of mean expression density of nuclei.

### Detection of β-casein, lactose secretion, and triglycerides

At 48 h post-transfection, the culture supernatant was collected for detection. β-casein, lactose secretion, and triglycerides were detected using an ELISA Kit for Casein Beta (CSN2) (New England Biolabs Inc., Beverly, Massachusetts, USA), a Lactose & D-Galactose (Rapid) Assay Kit (Megazyme, Bray Business Park, Bray, Ireland), and a Triglyceride (TG) GPO-POD Assay Kit (Applygen Tech Inc., Beijing, China) according to the manufacturer’s instructions. All experiments were performed in triplicate.

### Statistical analysis

The results were analyzed using SPSS 17.0 statistics software (Chicago, IL, USA). All results were expressed as the means ± standard deviation (SD) of separate experiments (n≥3). P values less than 0.05 were considered significant.

## Results

### Expression of miR-486 in bovine mammary gland tissues with different milk qualities

**Fig 1:**
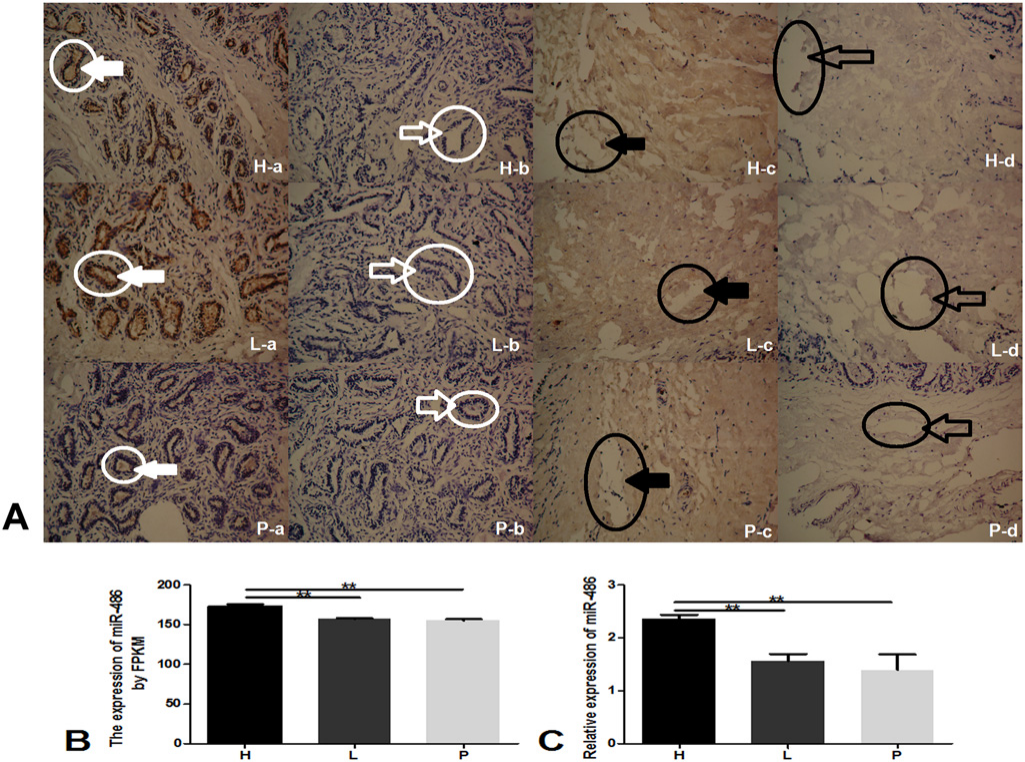
The expression of miR-486 in different milk qualities bovine mammary glands. A: ISH showing the localization of miR-486 in the mammary gland. H: lactation stage with high milk quality; L: lactation stage with low milk quality; P: pregnancy stage. (a) Mammary gland tissue incubated with a DIG-labeled locked nucleic acid probe against miR-486 (probe positive). And the white circle with solid white arrow indicates miR-486 positive probe in mammary gland tissue. (b) Mammary gland tissue incubated with a negative control probe (probe negative). And the white circle with feint arrow indicates miR-486 negative probe in mammary gland tissue. (c) Adipose tissue incubated with a DIG-labeled locked nucleic acid probe against miR-486 (fat positive). And the black circle with solid black arrow indicates miR-486 positive probe in adipose tissue. (d) Adipose tissue incubated with a negative control probe. And the black circle with feint black arrow indicates miR-486 negative probe in adipose tissue. B: The results of small RNA sequencing for miR-486 (fat negative). C: Analysis of the relative expression of miR-486 in bovine glandular tissue by qRT-PCR. n = three cows in every group. qRT-PCR reactions were performed in triplicate to detect miR-486. **P<0.01.

To examine whether miR-486 expression depends on the stage of mammary development specifically during the high-milk-quality lactation period (H) (Fig. 1A-1D), the low-milk-quality lactation period (L) (Fig. 1E-1H), and the pregnant period (P) (Fig. 1I-1L), we used *in situ* hybridization to evaluate the expression of miR-486 in bovine glandular tissue and adipose tissue (Fig. 1A). *In situ* hybridization using a DIG-labeled locked nucleic acid probe against miR-486 revealed that miR-486 was primarily expressed in bovine mammary glandular epithelium tissue (Fig. 1A: H-a, L-a, P-a) and was not expressed in bovine mammary adipose tissue (Fig. 1A: H-c, L-c, P-c).

Additionally, to determine if miR-486 expression was different between the high-milk quality and low-milk quality lactation periods we used a small RNA sequencing approach developed by our laboratory. And we found that the glandular expression of miR-486 was higher in high milk quality cows than in low milk quality cows (*P*<0.01) and pregnant cows (*P*<0.01). These results are shown in Fig. 1B. Furthermore, qRT-PCR analysis showed that the expression of miR-486 was higher in the H group than in the L (*P*<0.01) and P groups (*P*<0.01) (Fig. 1C).

### MiR-486 targets the PTEN gene

**Fig 2:**
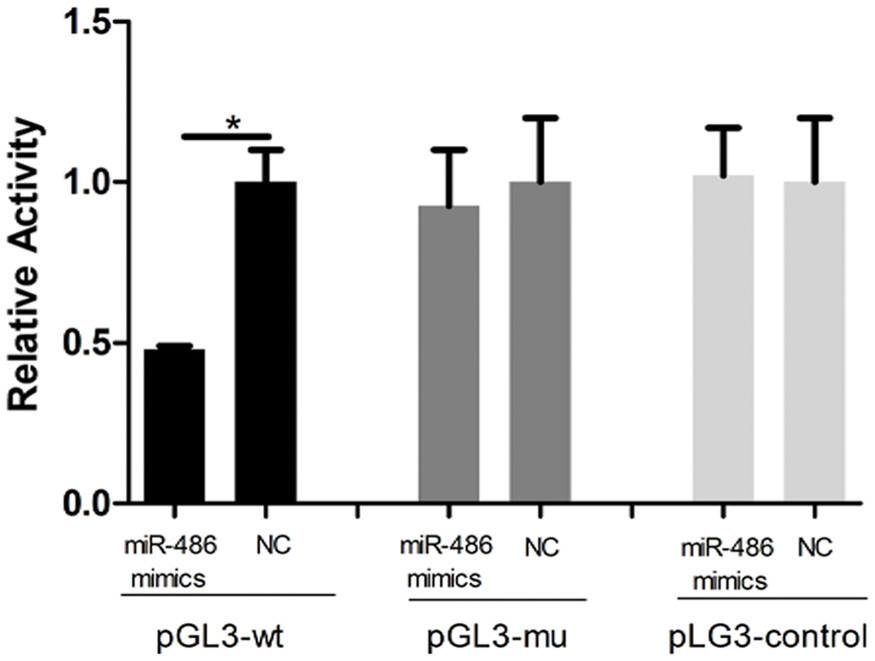
The PTEN 3’UTR is a target of miR-486. MiR-486 targets PTEN directly as measured by luciferase reporter assays, pGL3-wt: pGL3/PTEN and pGL3-mu: pGL3/PTEN/mut; pGL3-control: the empty vector, Luciferase activities of pGL3-wt are markedly decreased in cells transfected with miR-486 compared to those of reporter plasmids with mut or empty vectors. The data are the averages of at least three independent runs, and the values are the means±SD. *P<0.05 compared to the negative control.

MiR-486 is conserved in mammals and is the sole miRNA with no known family members [14]. Using the prediction software TargetScan6.2, we found that miR-486 target sites are present at nt 725–732 and nt 3,183–3,190 of the PTEN 3′UTR; these two sites are conserved in mammals. The 3′UTR of wild-type PTEN was linked to pGL3 Luciferase Reporter Vectors (Promega) and examined in HEK-293 cells that had been transfected with miR-486 mimics or a negative control. A significant decrease in reporter activity was observed when using the miR-486 mimics compared to the negative control. Transfection with miR-486 mimics had little effect on the luciferase activity of the mutant 3′UTR-reporter of PTEN compared with the negative control. Moreover, the luciferase signal of the pGL3-control vector with miR-486 was similar to that obtained with the negative control as shown in Fig. 2.

### MiR-486 inversely correlates with PTEN in BMECs

**Fig 3:**
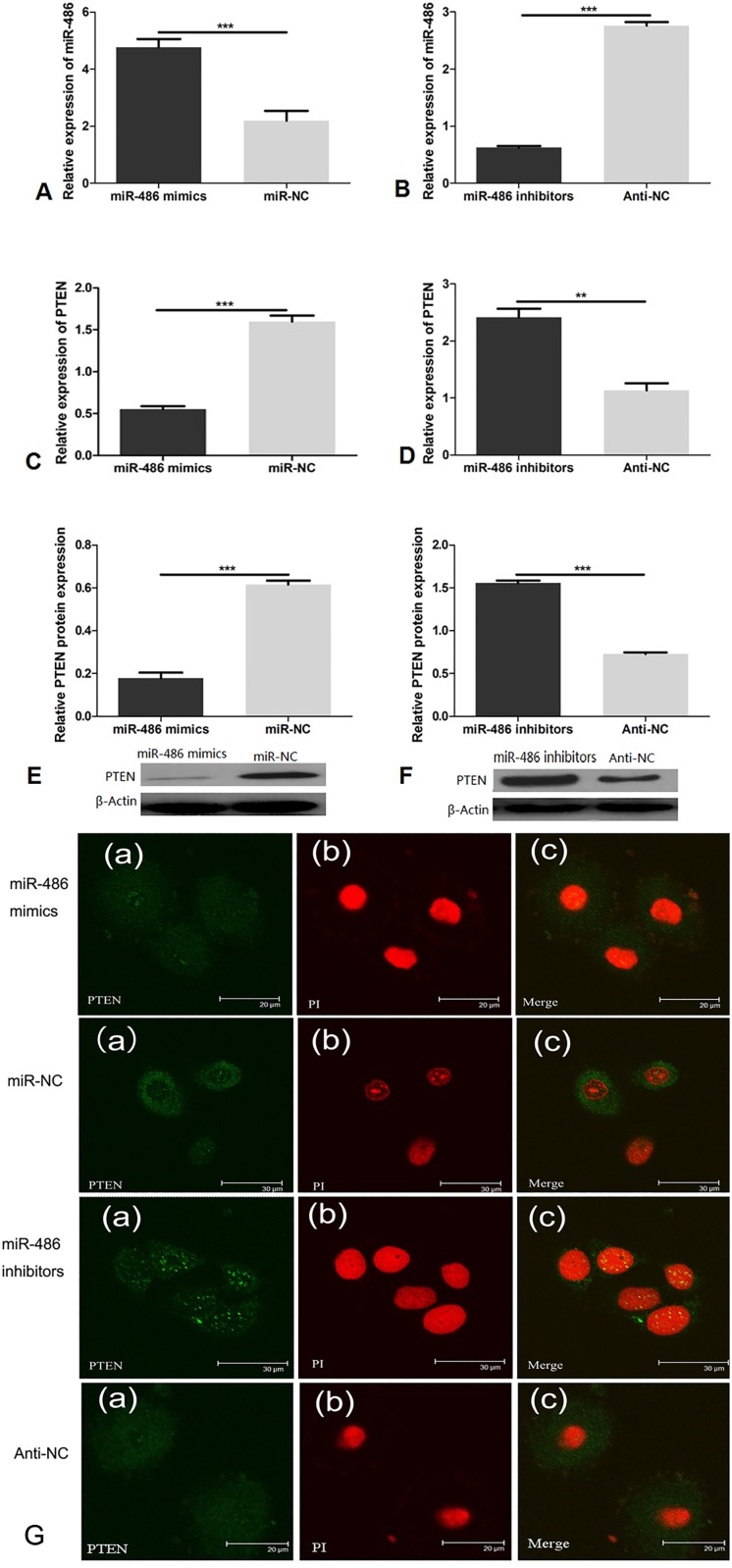
Transient transfection of BMECs with miR-486 mimics/inhibitor, led to changes in miR-486 and PTEN. A: qRT-PCR analysis of miR-486 expression in BMECs after transfection with miR-486 mimics or negative control (miR-NC). B: qRT-PCR analysis of miR-486 expression in BMECs after treatment with miR-486 inhibitors or the negative control (Anti-NC). C: qRT-PCR analysis of PTEN expression in BMECs after treatment with miR-486 mimics or negative control (miR-NC). D: qRT-PCR analysis of PTEN expression in BMECs after treatment with miR-486 inhibitors or negative control (Anti-NC). E: Western blotting analysis of PTEN protein expression in BMECs after treatment with miR-486 mimics or negative control (miR-NC). F: Western blotting analysis of PTEN protein expression in BMECs after treatment with miR-486 inhibitors or negative control (Anti-NC). All of the above experiments were performed in triplicate. G: Confocal microscopy analysis of PTEN expression in BMECs after treatment with miR-486 mimics (30 μM), negative control (miR-NC) (30 μM), miR-486 inhibitors (30 μM), or negative control (Anti-NC) (20 μM). (a): PTEN, (b): nuclear staining with propidium iodide, (c): merged images of (a) and (b). MiR-486 targets PTEN and represses the expression of PTEN mRNA and protein. The indicated values are the means±SD, ***P<0.0001.

We found that the expression of miR-486 increased after treatment with miR-486 mimics (Fig. 3 A, *P*<0.0001) and decreased after treatment with miR-486 inhibitors (Fig. 3 B, *P*<0.0001). To investigate whether miR-486 affects the regulation of the PTEN in mammary epithelial cells, we measured the relative expression of PTEN using qRT-PCR and western blotting. We found that the level of miR-486 changed concomitantly with the mRNA level of PTEN and that miR-486 inhibited the expression of PTEN (Fig. 3 C, Fig. 3 E, *P*<0.0001). Transfecting BMECs with a miR-486 inhibitor increased the expression of PTEN (Fig. 3 D, *P*<0.01; Fig. 3F, *P*<0.0001). We assessed the expression of PTEN after treatment with miR-486 mimics/inhibitors in BMECs using immunohistochemistry. We observed the expression of PTEN both in the cell nuclei and cytoplasm of mammary gland tissues (Fig. 3 G). A high expression of miR-486 repressed the expression of PTEN in mammary epithelium cell nuclei. However, a reduced expression of miR-486 stimulated PTEN expression in mammary epithelial cell nuclei. Thus, the data above suggest that miR-486 negatively affects PTEN.

### MiR-486 increases the expression of AKT and MTOR in bovine mammary epithelial cells

**Fig 4:**
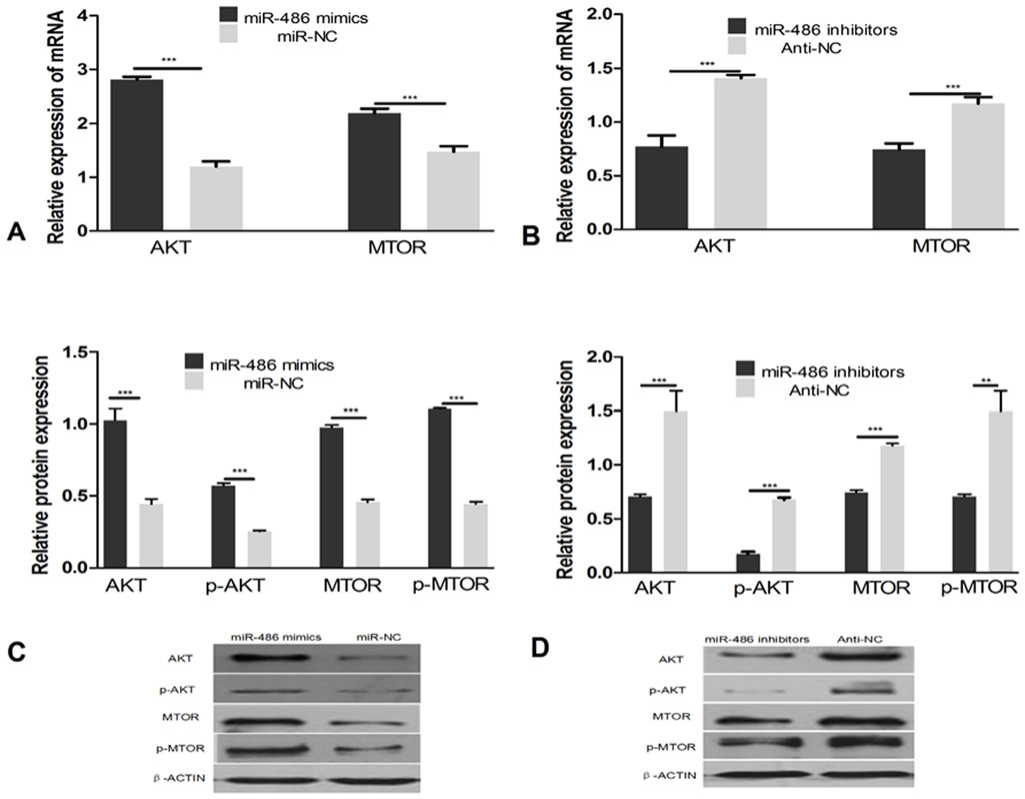
Expression of AKT and mTOR after the over-expression and inhibition of miR-486 in BMECs. A: mRNA level of AKT and mTOR after treatment with miR-486 mimics and negative control (miR-NC) in BMECs; B: mRNA level of AKT and mTOR during treatment with miR-486 inhibitors and negative control (Anti-NC) in BMECs; C: Protein level of AKT, p-AKT, mTOR and p-mTOR after transfected with miR-486 mimics and negative control (miR-NC) in BMECs; D: mRNA level of AKT and mTOR during treatment with miR-486 inhibitors and negative control (Anti-NC) in BMECs. Values are means±SD, **P<0.01, ***P<0.0001.

MiR-486 significantly repressed PTEN expression in cell culture and induced AKT and MTOR expression in BMECs. We observed that, compared to a negative control, the high-level expression of miR-486 stimulated the expression of AKT and MTOR (Fig. 4 A, *P*<0.0001). Moreover, the knockdown miR-486 repressed the expression of AKT and MTOR (Fig. 4 B, *P*<0.0001). The relative protein expression of AKT, phospho-AKT (p-AKT), MTOR, and phospho-MTOR (p-MTOR) increased as the expression of miR-486 was increased (Fig. 4 C, *P*<0.0001). However, decreasing the levels of miR-486 reduced the relative protein expression of AKT, p-AKT, MTOR, and p-MTOR (Fig. 4 D; *P*<0.0001, *P*<0.0001, *P*<0.01, respectively).

### Effect of miR-486 mimics/inhibitor on bovine mammary epithelial cell proliferation

**Fig 5:**
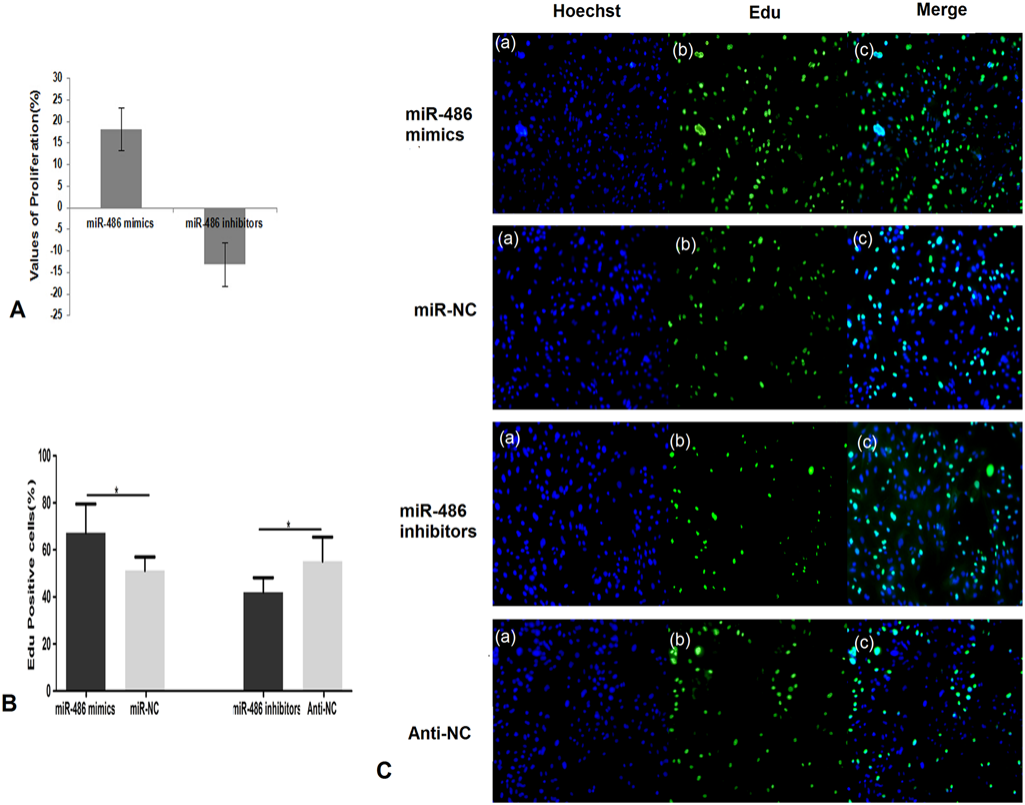
The effects of the miR-486 mimics and inhibitor on cell proliferation in BMECs. A: The CCK8 assay was performed at 48h after transfection of miR-486 mimics and miR-486 inhibitors; B: The percentage of EdU positive cells; C: EdU incorporation assays following transfected with miR-486 mimics, miR-486 inhibitors and their respective controls (miR-NC and Anti-NC) in BMECs for 48h. (a): all nuclear staining with Hoechst (100×), (b): nuclear staining with Edu (100×), (c): merged images of (a) and (b) (100×). Data were averages of at least three independent runs; Values are means±SD, *P<0.05.

To assess whether miR-486 affects the proliferation of bovine mammary epithelial cells, these cells were transfected with miR-486 mimics/inhibitor and analyzed using CCK-8 assays after 48 h. We measured the proliferation rate of the miR-486 mimics group and the miR-486 inhibitor group; miR-486 mimics increased the ability of the cells to proliferate, and the miR-486 inhibitor suppressed the ability of the cells to proliferate (Fig. 5 A). Additionally, we used the EdU incorporation assay to determine the role of miR-486 in the proliferation of BMECs. The percentage of Edu-positive cells was significantly higher in the miR-486 mimic-treated cells than in the negative control group (*P*<0.05). Additionally, the percentage of Edu-positive cells was lower in the miR-486 inhibitor group than in the negative control group (Anti-NC) (Fig. 5 B, *P*<0.05).

**Fig 6:**
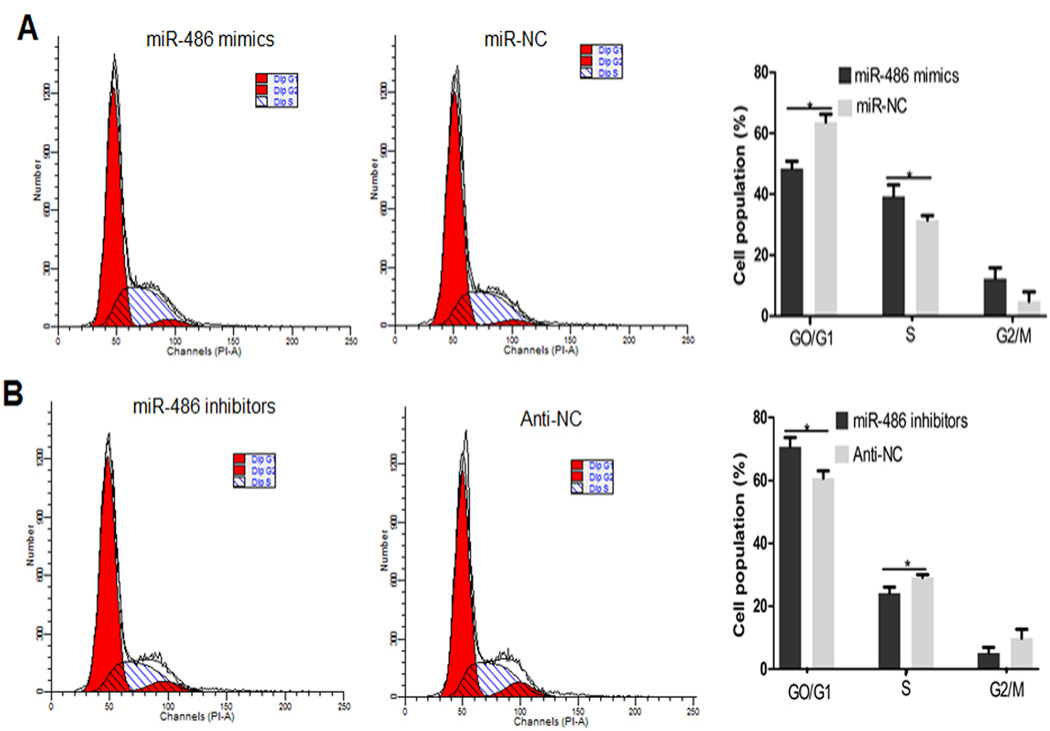
Changes in cell cycle distribution in BMECs due to miR-486 mimics and a miR-486 inhibitor. A: With the treatment of miR-486 mimics and negative control (miR-NC) in BMECs and stained with propidium iodide for flow cytometry; B: With the treatment of miR-486 inhibitors and negative control (Anti-NC) in BMECs and stained with propidium iodide for flow cytometry. Values are means±SD, *P<0.05.

Next, BMECs transfected with miR-486 mimics, inhibitor, or negative control were examined for changes in the cell cycle using flow cytometry analysis (Fig. 6). Transfection with miR-486 mimics resulted in an increased population of cells in the S phase and a decreased population of cells in G0/G1 compared to transfection with the negative control (Fig. 6 A, *P*<0.05). Furthermore, miR-486 inhibitors caused the opposite effect compared to the negative control (Anti-NC), (Fig. 6 B, *P*<0.05). The data above indicate that miR-486 is a negative modulator of the G1 to S transition.

### β-casein levels, lactose secretion, and triglyceride levels are upregulated by miR-486

**Fig 7:**
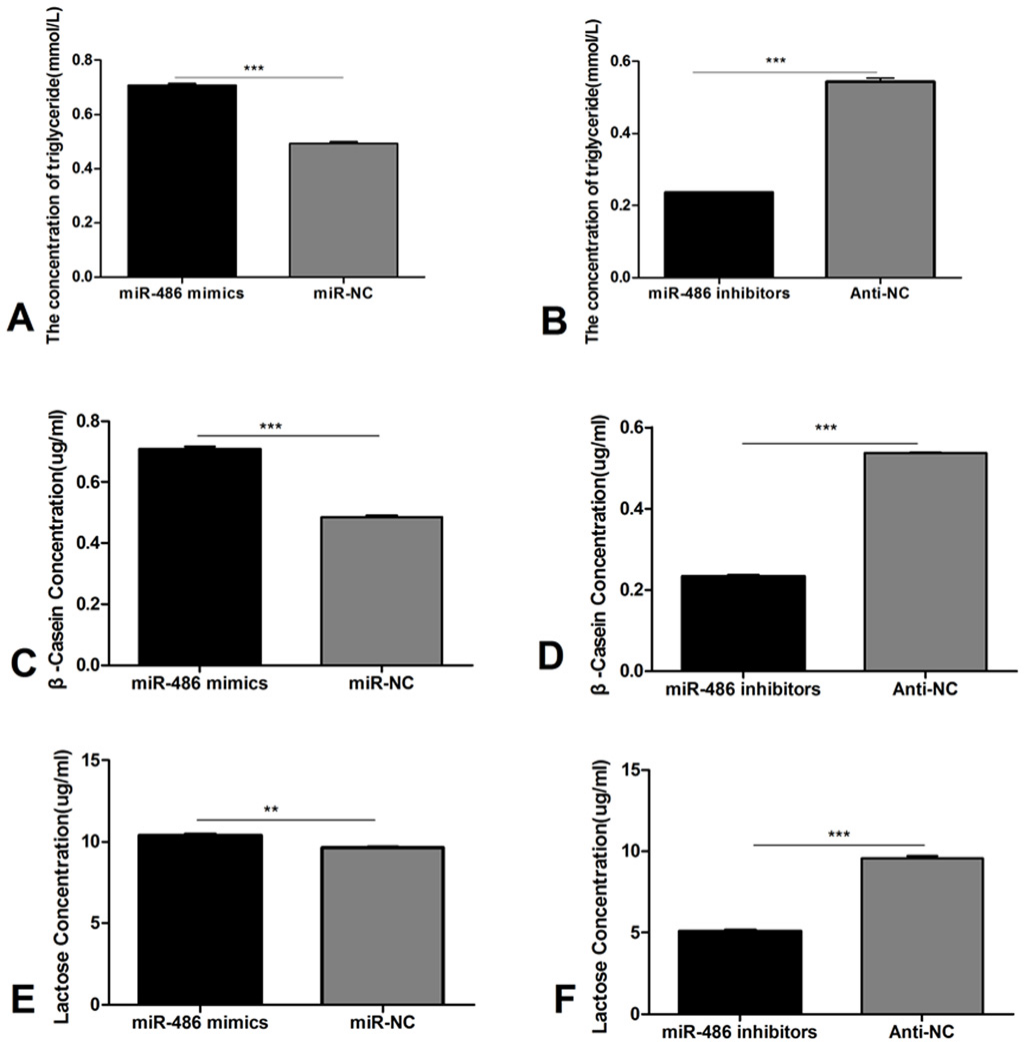
Analysis of secretion in mammary supernatants. A: Secretion of triglyceride following overexpressive miR-486, compared with negative control (miR-NC); B: Secretion of triglyceride following inhibiting miR-486, compared with negative control (Anti-NC); C: Secretion of β-casein following overexpressive miR-486, compared with negative control (miR-NC); D: Secretion of β-casein following inhibiting miR-486, compared with negative control (Anti-NC); E: Secretion of lactose following overexpressive miR-486, compared with negative control (miR-NC); F: Secretion of lactose following inhibiting miR-486, compared with negative control (Anti-NC). Data were averages of at least three independent runs; Values are means±SD, **P<0.01, ***P<0.0001.

Mammary epithelial cells synthesize and secrete proteins, lactose, and lipids. Thus, we examined the supernatant for changes in the levels these key components. The overexpression of miR-486 increased the concentrations of triglycerides, β-casein and lactose (Fig. 7 A, 7 C, and 7 E, *P*<0.0001; *P*<0.0001; *P*<0.001); conversely, inhibition of miR-486 suppressed the concentrations of triglycerides, β-casein and lactose (Fig. 7 B, 7 D, and 7 F, *P*<0.0001).

## Discussion

Currently, many miRNAs have been identified using algorithmic and experimental methods in animals, plants, and viruses. Many miRNAs have been found to play roles in cell differentiation, proliferation, apoptosis, development, tumorigenesis, and host-pathogen interactions [19–24]. Although experiments have been performed to identify the roles of an increasing number of miRNAs, few studies have been conducted to examine the role of miRNAs in the bovine mammary gland. Here, we present a study on the role played by miR-486 in the bovine mammary gland. This study shows that the tumor suppressor PTEN is negatively regulated by miR-486 via two specific sites (nt 725–732 and nt 3,183–3,190) within the 3`UTR. MiR-486 has been sparsely studied to date, and its relevance to the mammary gland has only begun to be elucidated. Using a small RNA sequencing approach, we observed that the levels of miR-486 were higher in lactation gland tissue during the high-quality milk stage than in the low-quality milk and pregnancy stages and that the expression of miR-486 was almost unchanged from the low-quality lactation stage to the pregnancy stage. We further validated this result using qRT-PCR and small RNA sequencing. *In situ* hybridization showed that miR-486 was found primarily in mammary gland tissue and not in adipose tissue. Therefore, we concluded that the post-transfection effects of miR-486 mimics or a miR-486 inhibitor affected mammary epithelial cells more than fat cells. The results were similar for almost all stages of bovine mammary gland development. Therefore, we believe that miR-486 is a key biomarker in bovine mammary gland epithelial cells.

Heterozygous PTEN mice were crossed with MMTV-wnt1 transgenic mice, resulting in the formation of breast tumors earlier in life than in the parental strains [25]. Some studies have examined the role of PTEN in normal mammary glands, but few studies have examined the secretion of these glands, which might prove that the tumor suppressor protein PTEN controls mammary gland development [17]. PTEN is commonly inactivated in human cancers and acts as a key regulator of the PIP3/AKT/mTOR pathway. One of study showed PTEN functions as an inhibitor during mammary gland development and lactation in dairy cows[26]. Indeed, our study confirmed that levels of AKT, phosphorylated AKT, mTOR and phosphorylated mTOR change in response to PTEN and that PTEN levels appeared to depend on miR-486 levels. The overexpression of miR-486 inhibited the expression of PTEN both at the mRNA and protein levels and thus increased the levels of AKT, phosphorylated AKT, mTOR and phosphorylated mTOR. PTEN is a well-known tumor suppressor in numerous cancers and normal tissues and regulates the AKT and mTOR signaling pathways. Our study provided information about the levels of nuclear PTEN. Bovine mammary epithelial cells overexpressing miR-486 had decreased PTEN expression in their nuclei; however, BMECs lacking miR-486 had increased PTEN levels. In general, despite its key role at the plasma membrane, PTEN is present in the nucleus of diverse cell types [27–28] (including tissue cells and cell lines [29–31]) and participates in many cellular processes that are relevant to tumorigenesis [32]. The regulation of PTEN nuclear localization is complex because PTEN plays many roles within the nucleus [17]. MiR-486 inhibited the expression of PTEN, and as same time cytoplasmic PTEN cellular staining towards nuclear staining, for PTEN/AKT/MTOR pathway plays prominent roles in development and lactation of mammary gland [26]. Here we suppose that miR-486 regulates expression of cytoplasmic PTEN, PTEN shift from cellular towards nuclear in mammary epithelial cells increase AKT/ MTOR pathway, and regulate mammary epithelial cells secretion of β-casein, triglyceride, and lactose, and plays a critical role in lactation related signaling pathways.

During the lactation period, the quality of milk that is secreted depends on milk fat (mostly triglyceride), milk protein (mostly β-casein) [33] and lactose. The results of this study show that triglyceride, β-casein and lactose levels were regulated by the transfection of miR-486 mimics or miR-486 inhibitors, indicating that miR-486 stimulated the lactation of milk fat, milk protein and lactose in the cow mammary gland.

The number and activity of mammary gland secreting cells in cows have been reported to affect the quality of milk [34]. We found that overexpressing miR-486 increased the amount of mammary epithelial cell proliferation as determined by CCK-8 assays. However, the inhibition of miR-486 showed the opposite effect. Edu incorporation experiments illustrated that miR-486 stimulated DNA proliferation by increasing the percentage of Edu-positive cells. Furthermore, our studies showed that miR-486 regulated the cell cycle of BMECs. MiR-486 mimics increased the population of BMECs in the G0/G1 phase and decreased the population of BMECs in the S stage. Therefore, we propose that miR-486 is a novel regulator that stimulates the proliferation of BMECs. The role of miR-486 in the cell cycle is an ongoing area of study.

## Conclusion

MiR-486 plays a key role in the mammary gland and directly downregulates the PTEN gene and affects downstream genes, such as AKT, mTOR, and β-casein, which play important roles in the mammary gland. Our data suggest that the repression of PTEN by miR-486 is associated with lactation and in this way increases AKT and mTOR. Additionally, miR-486 stimulates cell proliferation and increases some crucial secretory elements, such as β-casein, lactose, and lipids. Taken together, our findings indicate that miR-486 functions primarily in bovine mammary gland epithelium tissue and cells and promotes milk synthesis and secretion.
